# Computationally Efficient Implementation of a Novel Algorithm for the General Unified Threshold Model of Survival (GUTS)

**DOI:** 10.1371/journal.pcbi.1004978

**Published:** 2016-06-24

**Authors:** Carlo Albert, Sören Vogel, Roman Ashauer

**Affiliations:** 1 Eawag: Swiss Federal Institute of Aquatic Science and Technology, Dübendorf, Switzerland; 2 Environment Department, University of York, Heslington, York, United Kingdom; Universite de Montreal, CANADA

## Abstract

The General Unified Threshold model of Survival (GUTS) provides a consistent mathematical framework for survival analysis. However, the calibration of GUTS models is computationally challenging. We present a novel algorithm and its fast implementation in our R package, GUTS, that help to overcome these challenges. We show a step-by-step application example consisting of model calibration and uncertainty estimation as well as making probabilistic predictions and validating the model with new data. Using self-defined wrapper functions, we show how to produce informative text printouts and plots without effort, for the inexperienced as well as the advanced user. The complete ready-to-run script is available as supplemental material. We expect that our software facilitates novel re-analysis of existing survival data as well as asking new research questions in a wide range of sciences. In particular the ability to quickly quantify stressor thresholds in conjunction with dynamic compensating processes, and their uncertainty, is an improvement that complements current survival analysis methods.

This is a *PLOS Computational Biology* Software paper.

## Introduction

Survival analysis is an important tool in a wide range of scientific fields, including toxicology [1–4], epidemiology [5, 6], pharmacology [7], medical research [6, 8–10], and biology [11–13]. In the engineering world survival analysis is known as reliability theory [14, 15] whereas in the social sciences it is termed event history analysis [16, 17].

Common to these applications is the interest in the survival of individuals in response to a stressor. The assumptions underlying survival models have been reviewed recently and the General Unified Threshold model of Survival (GUTS) has been proposed as a consistent mathematical framework [4]. The GUTS framework has been developed primarily with environmental toxicology questions in mind and consequently it allows to model different dose metrics [18] and is a dynamic framework where toxicokinetic processes modify the dose metric and toxicodynamic processes result in the organisms’ response [4, 19, 20]. Typically, GUTS is used to model survival under chemical stress, for example under time-varying exposure [21] or to compare different organisms [22] and life-stages [23], however, stressors other than chemicals (e. g. starvation [24, 25]) can also be modelled. Often the parameters of GUTS are interpreted as reflecting the mechanisms affecting survival [4, 26].

One important consideration when modelling survival is the nature of death. Death can be viewed as deterministic on the level of the individual but stochastic on the level of the population: an individual dies immediately when its stressor tolerance threshold is exceeded, but all individuals of the population have different thresholds. This special case of GUTS is termed individual tolerance (GUTS-IT). In the other extreme case, death is stochastic on the level of the individual only: all individuals are supposed to have the same stressor tolerance threshold, and once the stressor exceeds this threshold all individuals respond with the same increased probability to die (GUTS-SD) [4]. The GUTS proper model provides a unification of both assumptions, however, it requires the calibration of four toxicodynamic parameters: the dominant (recovery) rate constant, the killing rate and two parameters describing the threshold distribution. As it can be difficult to estimate the four toxicodynamic parameters from biological data [22, 27], GUTS proper is often simplified to its two special cases, GUTS-IT and GUTS-SD [18, 21]. The special case models require the estimation of fewer toxicodynamic parameters and are therefore easier to apply, although the necessity to always use two models is rather unwieldy.

Furthermore, the evaluation of the likelihood function, for GUTS proper, requires two nested numerical integrations. As Bayesian inference requires many thousand likelihood evaluations, we have designed a very efficient algorithm for this evaluation.

Thus to enable the wider application of GUTS we present the first software package that allows this computationally non-trivial uncertainty quantification to be completed within a matter of minutes. The software is computationally efficient because we developed a novel algorithm, for the likelihood evaluation, and coded the underlying engine in C++. Further, our R package can be used for all three flavours of GUTS: GUTS proper, GUTS-SD and GUTS-IT.

## Design and Implementation

### The Algorithm

Before we present the algorithm, we give a brief review of the GUTS model (see [4], for more explanation). The time-dependent stressor, *C*(*t*), is assumed to cause a time-dependent damage, *D*(*t*), which is described by the linear differential equation

$$\dot{D}\left(t\right)=k_{e}\left(C\left(t\right)-D\left(t\right)\right)\;.$$ (1)

Parameter *k*_*e*_ quantifies the slowest process that leads to the recovery of the organism, and we will henceforth refer to it as the *dominant rate constant*. The damage *D*(*t*) is the same, for all individuals. However, the individuals are assumed to have different thresholds, beyond which the damage increases their probability to die. Thus, the model combines two sources of stochasticity, at the individual and at the population level. At the individual level, death is considered a stochastic event, whose probability increases linearly with the damage, once it exceeds a certain threshold. At the population level, this threshold is assumed to vary stochastically over the whole population.

The *hazard rate*, *h*_*z*_(*t*), of an individual with threshold *z* is determined by the formula

$$h_{z}\left(t\right)=k_{k}\text{max}\left(D\left(t\right)-z,0\right)+h_{b}\;,$$ (2)

where *k*_*k*_ is called *killing rate* and *h*_*b*_ is the *background mortality rate*. The *hazard rate*, in turn, determines the individual’s probability to survive until time *t*, *S*_*z*_(*t*), via the linear differential equation

$${\dot{S}}_{z}\left(t\right)=-h_{z}\left(t\right)S_{z}\left(t\right)\;.$$ (3)

Finally, each individual is assumed to draw its *z* from a distribution, *f*_***θ***_(*z*), on the positive real axis. Hence, the parameter vector of the model reads as ***θ*** = (*h*_*b*_, *k*_*e*_, *k*_*k*_, …), where the additional arguments are supposed to determine the distribution *f*_***θ***_(*z*).

Typically, *f*_***θ***_(*z*) is a member of a two-parameter family of distributions, such as the lognormal family. In these cases, GUTS has four toxicodynamic parameters, *k*_*e*_, *k*_*k*_, as well as the mean and the standard deviation determining the lognormal distribution. These are GUTS proper models. GUTS-SD and GUTS-IT are limiting cases of GUTS proper. In the first case, the standard deviation is zero, which means that all individuals have the same threshold given by the mean of the distribution. In the latter case, *k*_*k*_ is infinite, which means that individuals die immediately once their individual threshold is exceeded. Note that eq (2) may be viewed as a special case of Aalens additive model [28].

Combining eqs (2) and (3), we find that the probability, for an arbitrarily chosen member of the population, to survive until time *t* is given by the formula

$$S_{\theta }\left(t\right)=\int \text{exp}\left(-k_{k}\int_{0}^{t}\text{max}\left(D\left(\tau \right)-z,0\right)d\tau -h_{b}t\right)f_{\theta }\left(z\right)dz\;.$$ (4)

Let **y** = (*y*_0_, *y*_1_, …, *y*_*n*_) denote a time series of survivors, counted at times (*t*_0_ = 0, *t*_1_, …, *t*_*n*_), and set *y*_*n*+1_ = 0. Then, the logarithm of the likelihood, *f*(**y** **|** ***θ***), of the model output **y** given the parameters ***θ*** is, up to ***θ***-independent terms, given by the formula

$$\text{ln}f\left(y|\theta \right)=\sum_{i=1}^{n+1}\left(y_{i-1}-y_{i}\right)\text{ln}\left(S_{\theta ,i-1}-S_{\theta ,i}\right)\;,$$ (5)

where we have set *S*_***θ***, *i*_ = *S*_***θ***_(*t*_*i*_) and *S*_***θ***, *n*+1_ = 0. The index *n*+1 refers to the time-point at infinity, where the survival probability is zero.

The calculation of the log-likelihood requires two nested numerical integrations (see eq (4)), and, therefore, requires introducing two large numbers, *N* and *M*. The former counts the number of sample or discretisation points on the threshold axis and the latter counts the number of discretisation points on the time axis. Our algorithm is of the order O(N)+O(M). It is based on the approximation

$$\begin{array}{ll} S_{i} & =\;\int \text{exp}\left[-k_{k}\int_{0}^{t_{i}}\text{max}(0,D(\tau )-z)d\tau -h_{b}t_{i}\right]f_{\theta }(z)dz\\ & \approx \frac{1}{N}\sum_{j=1}^{N}\text{exp}\left[-k_{k}\Delta \tau \sum_{D_{l}>z_{j}}\left(D_{l}-z_{j}\right)-h_{b}t_{i}\right]\\ & =\frac{1}{N}e^{-h_{b}t_{i}}(e^{-k_{k}\Delta \tau (e_{N}-z_{N}f_{N})}+e^{-k_{k}\Delta \tau (e_{N}+e_{N-1}-z_{N-1}(f_{N}+f_{N-1}))}+\ldots \\ & +e^{-k_{l}\Delta \tau (e_{N}+\ldots +e_{1}-z_{1}(f_{N}+\ldots +f_{1}))}), \end{array}$$ (6)

for an ordered sample *z*_1_ < … < *z*_*N*_ from *f*_***θ***_(*z*), and with *D*_*l*_ = *D*(*τ*_*l*_) on a grid *τ*_0_ < … < *τ*_*M*−1_. The inner sum in the second line is restricted to all *D*_*l*_, for which *τ*_*l*_ < *t*_*i*_, and we have set Δ*τ* = *t*_*n*_/*M*. Furthermore,

$$e_{j}=\sum_{z_{j}<D_{l}<z_{j+1}}D_{l}\;,$$ (7)

and

$$f_{j}=♯\left\{D_{l}|z_{j}<D_{l}<z_{j+1}\right\}\;,$$ (8)

where ♯ indicates counting the number of elements in the set, for 1 ≤ *j* ≤ *N* (set *z*_*N*+1_ = ∞).

The algorithm for the calculation of eq (5) reads as follows:

**Step 1:** Draw *N* thresholds from *f*_***θ***_(*z*) and order them *z*_1_ < ⋯ < *z*_*N*_.
**Step 2:** Refine the grid *t*_0_ < … < *t*_*n*_ to a fine grid *τ*_0_ < ⋯ < *τ*_*M*−1_.
**Step 3:** Set *i* = 0.
**Step 4:** Solve eq (1), for *t*_*i*_ ≤ *τ*_*l*_ ≤ *t*_*i*+1_, using equation
  $$\begin{array}{ll} D_{l}=D(\tau_{l}) & =D\left(s_{k}\right)e^{-k_{e}\left(\tau_{l}-s_{k}\right)}\\ & +C_{k}\left(1-e^{-k_{e}\left(\tau_{l}-s_{k}\right)}\right)\\ & +\frac{C_{k+1}-C_{k}}{s_{k+1}-s_{k}}\left(\tau_{l}-s_{k}-k_{e}^{-1}+k_{e}^{-1}e^{-k_{e}\left(\tau_{l}-s_{k}\right)}\right), \end{array}$$ (9)
  for *s*_*k*_ ≤ *τ*_*l*_ ≤ *s*_*k*+1_. Here, we apply a linear interpolation, for *C*(*t*), between concentrations *C*_*k*_, measured at time points *s*_*k*_.
**Step 5:** Update eqs (7) and (8), for 1 ≤ *j* ≤ *N*. (This can be done in time O(1), for each *D*_*l*_).
**Step 6:** Calculate *S*_*i*_ using the recursion:
  $$F_{j}\;=\;F_{j+1}+f_{j},$$ (10)
  $$E_{j}\;=\;E_{j+1}+e_{j},$$ (11)
  $$S_{i,j}\;=\;S_{i,j+1}+e^{-k_{k}\Delta \tau (E_{j}-F_{j}z_{j})},$$ (12)
  for *j* = *N* − 1, …, 1 and with *S*_*i*, *N*_ = *e*^−*k*_*k*_ Δ*τ*(*E*_*N*_ − *F*_*N*_*z*_*N*_)^ and *F*_*N*_ = *f*_*N*_, *E*_*N*_ = *e*_*N*_. Then,
  $$S_{i}=\frac{1}{N}e^{-h_{b}t_{i}}S_{i,1}\;.$$ (13)
**Step 7:** Increment *i* and go to step **4**.
**Step 8:** Calculate the log-likelihood function according to eq (5).

Depending on the threshold distribution, the above algorithm can be made more efficient through importance sampling. That is, instead of sampling from *f*_***θ***_(*z*) we sample from distribution *g*_***θ***_(*z*) and correct with weights:

$$\begin{array}{ll} S_{i} & =\int^{\text{​}}\text{exp}\left[-k_{k}\int_{0}^{t_{i}}\text{max}\left(0,D\left(\tau \right)-z\right)d\tau -h_{b}t_{i}\right]f_{\theta }\left(z\right)dz\\ & =\int^{\text{​}}\text{exp}\left[-k_{k}\int_{0}^{t_{i}}\text{max}\left(0,D\left(\tau \right)-z\right)d\tau -h_{b}t_{i}+\text{ln}\left(f_{\theta }\left(z\right)/g_{\theta }\left(z\right)\right)\right]g_{\theta }\left(z\right)dz. \end{array}$$ (14)

The associated algorithm is then the same as above, except that we generate an ordered sample from *g*_***θ***_(*z*) and replace expression

$$e^{-k_{k}\Delta \tau \left(E_{j}-F_{j}z_{j}\right)}$$

by

$$e^{-k_{k}\Delta \tau \left(E_{j}-F_{j}z_{j}\right)+\text{ln}\left(f_{\theta }\left(z_{j}\right)/g_{\theta }\left(z_{j}\right)\right)}\;.$$

Furthermore, the vector of survival probabilities, *S*_*i*_, has to be normalised through element-wise division by the first unnormalised element of the vector.

If *f*_***θ***_(*z*) is the lognormal distribution, we recommend using a log-uniform distribution covering the highest probability region of *f*_***θ***_(*z*), and replacing the sample by a grid. More precisely, we set $z_{j}=e^{x_{j}}$, where {*x*_*j*_} describes an equidistant grid on the interval [*μ* − 4*σ*, *μ*+4*σ*], where

$$\mu =\text{ln}\left(m\right)-\frac{1}{2}\sigma^{2}\;,\;\sigma^{2}=\text{ln}\left(1+\frac{s^{2}}{m^{2}}\right)\;,$$ (15)

with *m* and *s*^2^, respectively, the mean and variance of the lognormal distribution. The weights become, up to an irrelevant ***θ***-independent term,

$$\text{ln}\left(f_{\theta }\left(z_{j}\right)/g_{\theta }\left(z_{j}\right)\right)=-\frac{1}{2}\frac{{\left(\mu -\text{ln}\left(z_{j}\right)\right)}^{2}}{\sigma^{2}}\;.$$ (16)

### Implementation in the R Package GUTS

The GUTS algorithm is implemented in the R package GUTS [29, 30], current version 1.0.0). R [31] is an open source software environment for statistical computing that provides a wide range of procedures for data manipulation, data analysis, simulation, modelling and producing graphics. R packages are extensions contributed by members of the R community to add functionality to the R environment.

The R package GUTS is such an extension, and it contains a setup function and functions to calculate the survival probabilities and the associated logarithm of the likelihood, respectively. In order to achieve high speed, the actual engine for the calculation of the survival probabilities and the associated logarithm of the likelihood is written in C++ and exposed to R through the deployment of Rcpp [32, 33]. The engine cannot be called directly but through the use of two wrapper functions. The function for the calculation of the log-likelihood is typically used in a parameter estimation routine, while the function for the survival probabilities can be used to make predictions. Both functions update the GUTS object directly, but also return the logarithm of the likelihood or the vector of survival probabilities, respectively. The help file of the package contains a detailed description of the package functions, their arguments and use (R command help(“GUTS”)).

The R package GUTS allows for the realisation of two models, the full model (GUTS Proper) and the individual tolerance model (GUTS-IT). In addition, the stochastic death model (GUTS-SD) can be achieved through the use of the delta distribution with model GUTS Proper. If the thresholds are sampled from the lognormal distribution (the default) and the full model (GUTS Proper, also default) is applied, 5 parameters are required:

- hb: background mortality rate
- ke: dominant rate constant
- kk: killing rate
- mn: mean of the threshold distribution
- sd: standard deviation of the threshold distribution

For the delta distribution, no standard deviation must be provided, and for the model GUTS-IT, the killing rate must be omitted. The number of parameters is checked according to the distribution and model. A wrong number of parameters invokes an error. However, improper parameter values (e.g., negative values) invoke a warning resulting in the vector of parameters and the vector of survival probabilities being set to NA, and the logarithm of the likelihood being set to -Inf.

For testing and demonstration purposes, the package also provides a data set, “diazinon”. Pulsed toxicity tests with the freshwater crustacean *Gammarus pulex* and diazinon, an organophosphate insecticide, were carried out to measure survival through time under repeated pulsed exposure with variable recovery phases between pulses. Exposure concentrations were measured frequently and survival was observed daily. The dataset contains the results from three different experiments (exposure scenarios), where each experiment started off with 70 alive individuals. For more details see [34].

The R package GUTS is (like R) licensed under GPL-2 and freely available from CRAN (http://CRAN.R-project.org, users should employ the package installation routines available in R). The package also includes a manual page with detailed information about the functions and their arguments.

## Practical Application Example

A typical application scenario of the R package GUTS comprises creating proper GUTS objects from data, performing the parameter estimation, computing the parameter uncertainty, and making probabilistic predictions as well as validations with new data. We present such a scenario using example data to model GUTS Proper with thresholds from the lognormal distribution. During our presentation we make use of self-defined wrapper functions, which serve to keep the actual workflow clear and simple. A complete ready-to-run script containing a detailed explanation of the code and functions can be found in the supplementary information.

### Read Data and Create GUTS Objects

After installing and loading all required packages, we read in data from experiments. For convenience, it is best to prepare a well-formatted text file and then use our wrapper function ga_read_list(). If, for instance, the data from [34] should be read in, the file must be formatted as follows:

# Gammarus pulex exposed to diazinon

C1:102.65,97.59,0,0,103.88,98.19,0,0,0,0

C2:100.78,106.32,0,0,103.56,95.82,0,0,0

C3:100.6,94.61,0,0,100.58,96.51,0,9.85

Ct1:0,1.02,1.03,2.99,3.01,4.01,4.02,11.01,18.01,22.01

Ct2:0,1.02,1.03,8,8.01,9,9.01,15,22.01

Ct3:0,1.02,1.03,16,16.01,17,17.01,22.01

y1:70,66,61,55,31,31,29,26,24,22,21,19,17,14,14,13,11,11,10,9,8,8,8

y2:70,65,59,56,54,50,47,46,46,40,23,22,22,21,18,17,17,13,13,13,11,11,11

y3:70,65,59,55,53,51,48,46,46,46,44,41,40,40,40,39,38,36,33,28,24,23,19

yt1:0,1,2,3,4,5,6,7,8,9,10,11,12,13,14,15,16,17,18,19,20,21,22

yt2:0,1,2,3,4,5,6,7,8,9,10,11,12,13,14,15,16,17,18,19,20,21,22

yt3:0,1,2,3,4,5,6,7,8,9,10,11,12,13,14,15,16,17,18,19,20,21,22

The first line shows a comment, which is ignored when reading in. Each data line starts with a variable name (e.g., C1 for the first concentration vector) followed by a colon, the actual data separated by commas, and each data vector terminated by a newline. The C and y vectors denote, respectively, exposure concentrations [nmol/l] and survival counts. The Ct and yt vectors denote the time points at which these vectors are measured [day]. As our algorithm assumes a linear interpolation between concentrations, the values have been chosen such that pulses of approximately rectangular shape are achieved.

Having such a plain text file created in the working directory under the file name, say, Data_Gp_Diazinon.txt, it can then be read in:

*diazinon <- ga_read_list(“Data_Gp_Diazinon.txt”)*

However, for testing and demonstration purposes, the diazinon data is included in the R package GUTS. We can load this data directly and create a list of 3 GUTS objects:

*data(diazinon)*

*guts_objects <- list(*

* guts_setup(C = diazinon[[“C1”]], Ct = diazinon[[“Ct1”]],*

*  y = diazinon[[“y1”]], yt = diazinon[[“yt1”]]),*

* guts_setup(C = diazinon[[“C2”]], Ct = diazinon[[“Ct2”]],*

*  y = diazinon[[“y2”]], yt = diazinon[[“yt2”]]),*

* guts_setup(C = diazinon[[“C3”]], Ct = diazinon[[“Ct3”]],*

*  y = diazinon[[“y3”]], yt = diazinon[[“yt3”]])*

*)*

Note, that all other arguments of the setup function (guts_setup()) already default to modelling GUTS-Proper (i.e., dist = “lognormal”, model = “Proper”, N = 1000, M = 10000) and are therefore omitted. However, for modelling GUTS-SD, set dist = “delta” and model = “Proper”, and for modelling GUTS-IT, set dist = “lognormal” and model = “IT”. In order to inspect the content of the first GUTS object in the list, use the print command, print(guts_objects[[1]]).

### Bayesian Parameter Estimation

The parameter estimation is achieved through the use of an optimisation routine to find good starting parameters, and the use of a MCMC routine, for sampling the parameter posterior distribution. We define the evaluation function logposterior(), which is the sum of the log-likelihood and the log-prior:

*logposterior <- function(pars) {*

* if (any(is.na(pars), is.infinite(pars),*

*  (pars<0), (pars[“kk”]>30))) {*

*  return(-Inf)*

* }*

* ret <- sum(sapply(guts_objects,*

*  function(obj) guts_calc_loglikelihood(obj, pars)))*

* return(ret)*

*}*

We have chosen uniform priors, for all parameters, with lower bounds equal to zero. The upper bounds are assumed sufficiently large so that they do not affect the posterior significantly. As the posteriors of all parameters except *k*_*k*_ decay sufficiently fast, no upper bounds need to be specified, for those parameters. The posterior of parameter *k*_*k*_, however, seems to exhibit a fat tail, which occasionally leads to divergent Markov chains. For this parameter, we specify a prior upper bound *k*_*k*_ < 30*l*/(*day* *nmol*) that is large enough to be practically indistinguishable from the IT regime *k*_*k*_ = ∞.

The function logposterior() takes a vector of parameters as its only argument. If the parameter vector lies outside the prior range, logposterior() returns -Inf; otherwise it applies a vector-wise calculation of the logarithm of the likelihood to the GUTS objects (sapply()) and returns the sum.

To find good starting parameters, we use an R implementation of the “Hooke-Jeeves derivative-free minimisation algorithm”. The optimiser hjkb() is included in the package dfoptim [35]. We then define a start vector as well as its lower and upper bounds needed during the optimisation.

*library(“dfoptim”)*

*pars_start <- c(0.05, 0.1, 3, 20, 10)*

*names(pars_start) <- c(‘hb’, ‘ke’, ‘kk’, ‘mn’, ‘sd’)*

*optim_result <- hjkb(pars_start, logposterior, lower = rep(0, 5),*

* upper = c(1, 1, 30, 40, 20), control = list(maximize = TRUE))*

Warnings invoked by the GUTS package functions can be inspected using the command warnings() (for their meaning, see the help file and section Implementation in the R Package GUTS). However, these warnings do not affect the optimisation and can, therefore, be safely ignored.

The result of the optimisation routine is inspected using the print function (for an in-depth description of the output consult the manual page of hjkb()):

*print(optim_result)*

$par

    hb     ke     kk      mn     sd

 0.05473022  0.09215698  1.80652237  15.63446045  6.01160431

$value

[1] -570.6315

$convergence

[1] 0

$feval

[1] 14479

$niter

[1] 19

Note that choosing reasonable parameter bounds can hardly be automatised and thus relies on the expert knowledge about common parameters, for the respective types of data and experiment. If expert knowledge suggest different parameters and bounds, the vectors above need to be adjusted [26].

The posterior parameter distribution is sampled using the robust adaptive Metropolis sampler implemented in the R package adaptMCMC [36] with the parameters from the optimisation serving as starting values. The function MCMC() automatically adapts the covariance of the jump distribution to achieve a user-defined acceptance rate (here: 0.4). As a starting value, for the covariance of the jump distribution, we simply use a diagonal one with 10% of the initial parameter values as standard deviations. In order to prevent degeneracy of the matrix (in case the optimiser returns zero, for certain parameters), the matrix is altered by adding a small positive number to the diagonal. Note that, although the R package GUTS is very fast, the MCMC may take some minutes depending on the number of iterations chosen (argument n of the function MCMC()) and the hardware used (on our testing MacBook Pro with a 4 core Intel i7 processor 50,000 iterations took about 3 minutes). Like in the optimisation routine, warnings can occur and can be ignored (see above).

*library(“adaptMCMC”)*

*mcmc_pars <- optim_result$par*

*mcmc_sigma <- diag((mcmc_pars/10)^2 + .Machine$double.eps)*

*mcmc_result <- MCMC(p = logposterior, init = mcmc_pars,*

* scale = mcmc_sigma, adapt = 20000, acc.rate = 0.4, n = 50000)*

### Visualisation of the Posterior Distribution

**Fig 1:**
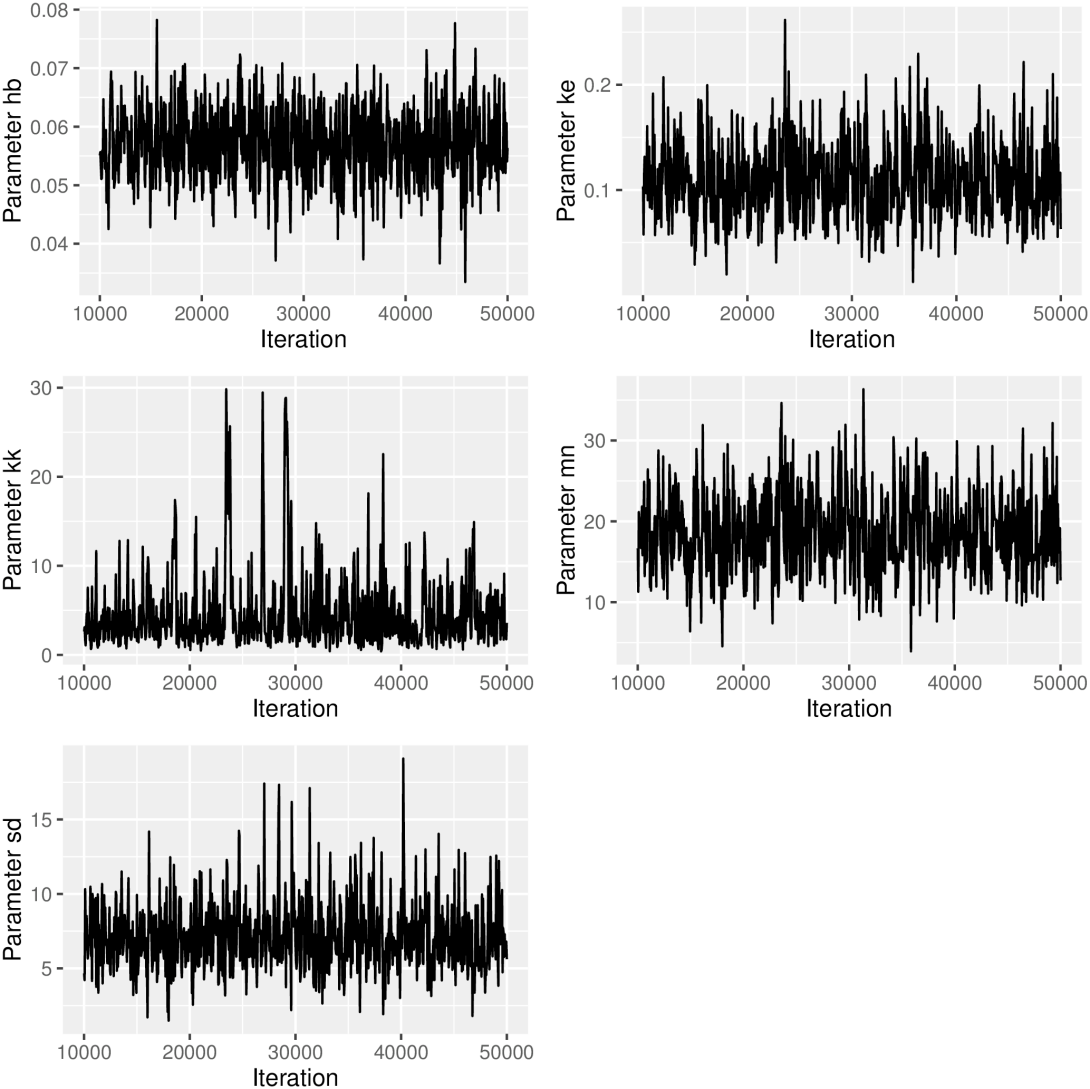
Chains of the parameters’ posterior marginals computed by the MCMC.

After the MCMC has finished without errors, it is necessary to inspect the chains and check whether they have converged. Automatised checks are available (e.g., through using CODA [37]), however, here we create a plot from the chains of the parameters’ posterior marginals and check the chains visually (see Fig 1).

*plot_chains <- ga_plot_chains(data = mcmc_result$samples,*

* from = 10000, steps = 50)*

After cutting off the first 10,000 sample points, the chains show a reasonable mixing and no signs of burn-in or the adaptation phase of the algorithm. We have chosen the function MCMC() from package adaptMCMC because it is largely self-tuning and therefore easy to use. An adaptation phase of 20,000 sample points seems to be sufficient in most cases. Nevertheless, it is important to keep in mind that MCMC() calls a stochastic algorithm, which may fail to adapt, even for the data set we are using. If that happens, running the algorithm a second time usually suffices. If not, the adaptation phase might have to be enlarged and the chain might have to be run for a longer time.

**Fig 2:**
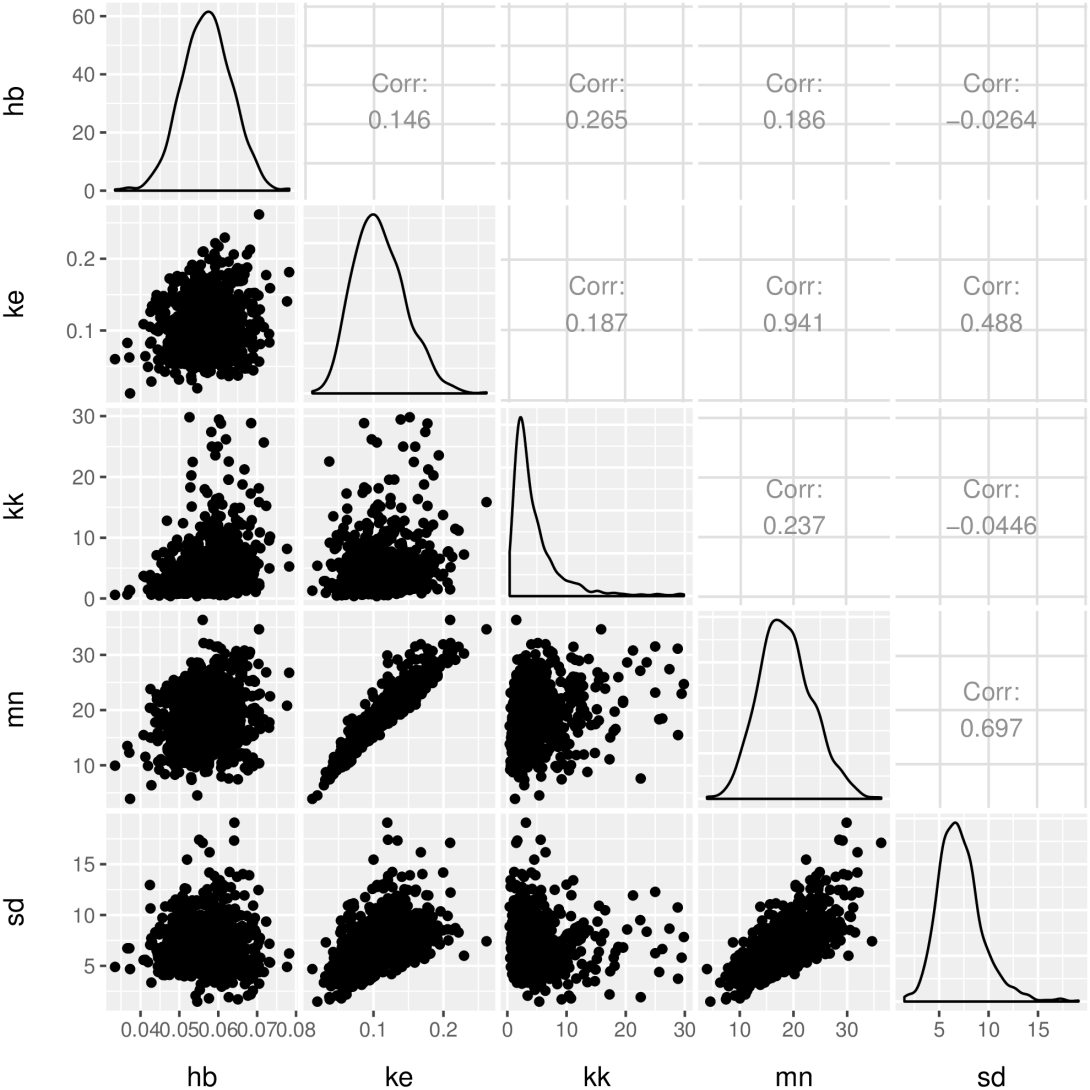
Correlations between the parameter posterior samples computed by the MCMC.

Next, we create a correlation plot, for the posterior parameter sample, computed by the MCMC. The parameters for our example co-vary as shown in Fig 2.

*plot_corrs <- ga_plot_correlations(data = mcmc_result$samples,*

* from = 10000, steps = 50)*

The strongest correlation is observed between the threshold mean (*mn*) and the dominant rate constant (*k*_*e*_), which can be understood from eqs (1) and (2). Strong parameter correlations could be viewed as indicators of over-parametrised models, however the equations of GUTS represent our understanding of the processes determining survival under stress. As such the model parameters have a mechanistic interpretation, which would be partially lost if reducing the model. Furthermore, reducing GUTS to fewer parameters would introduce additional strong assumptions and so GUTS would loose its generality. For example, disposing of the threshold parameter would imply the assumption that any infinitely small dose of the stressor will result in an increased hazard rate (see also [4]). An important insight from Fig 2 is that survival predictions must account for the correlation between parameters to properly account for parametric uncertainty.

### Quantification of Parameter Uncertainty

To compute the uncertainty of each of the parameters, we calculate adequate quantiles from the posterior samples. Together with the maximum of the posterior distribution, these quantiles are then tabulated.

*tab_quant <- ga_tab_quantiles(data = mcmc_result$samples,*

* log.p = mcmc_result$log.p, from = 10001)*

*print(tab_quant)*

     maxpost   q0.025      q0.5    q0.975

hb  0.05473022 0.04497855  0.05671463  0.06952769

ke  0.09215698 0.04676900  0.10578718  0.18960682

kk  1.80652237 0.86402773  3.22829131   17.27880308

mn 15.63446045  9.59333778  18.33559994    28.89464348

sd  6.01160431 3.44263752  6.86731117    12.31967439

**Fig 3:**
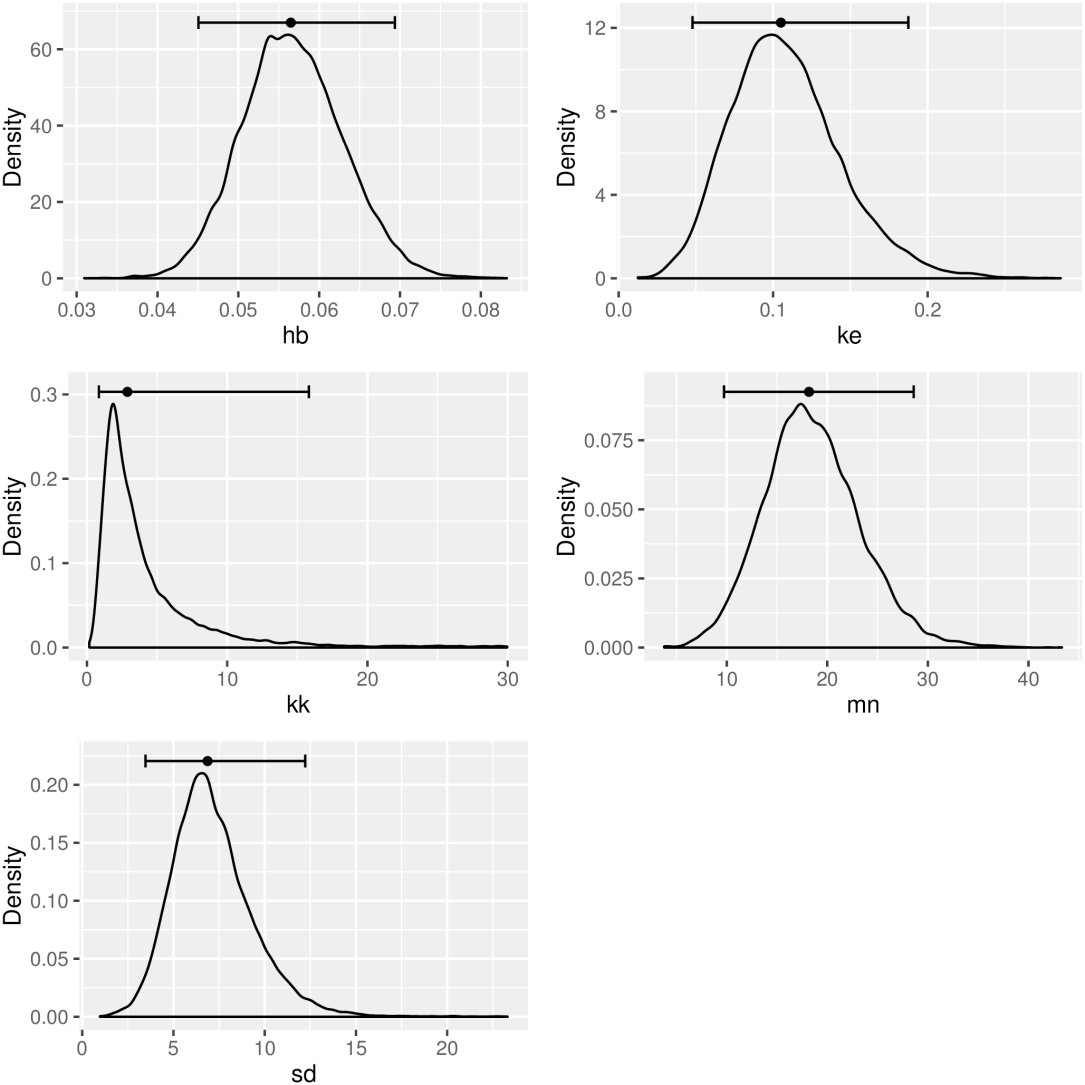
Densities and uncertainty (quantiles) of the parameter posteriors.

To inspect the distribution and the uncertainty visually, we plot the densities of each of the parameters. Fig 3 shows the densities, and each plot contains a horizontal line indicating the uncertainty quantiles (the median is always added).

*plot_dens <- ga_plot_densities(data = mcmc_result$samples)*

### Probabilistic Prediction and Validating the Model With New Data

After calibrating our model with real data, we use it for probabilistic predictions. We demonstrate how to make probabilistic predictions (without survival data) and how to validate these predictions against measured survival data. In both cases we use fictional (“fake”) data, for demonstration purposes.

The data must contain concentrations and concentration time points, but also the time points at which we want to make predictions. Furthermore, we need to specify the initial number of individuals (100, in our example). This number is set in the first element of the vector of survivor counts (y). Unless we have validation data, the remaining values are not needed and set to an arbitrary value (0, in our example).

*g_obj_new <- list(*

* guts_setup(C = c(99.97824, 0, 103.88, 0, 0, 103.56, 0, 0,*

*   100.58, 96.51, 0, 2.35724),*

*  Ct = c(0, 1.03, 3.01, 4.02, 8, 8.01, 15, 16,*

*   16.01, 17, 18.01, 22.01),*

*  y = c(100, rep(0, 22)),*

*  yt = 0:22),*

* guts_setup(C = c(101.343, 99.5066, 0, 98.19, 95.82, 0, 0,*

*   0, 3.283),*

*  Ct = c(0, 1.02, 2.99, 4.01, 9, 9.01, 11.01,*

*   17.01, 22.01),*

*  y = c(100, rep(0, 22)),*

*  yt = 0:22)*

*)*

We tabulate the predictions and save the result in the R list object tab_pred. We use the command head() to print the first 6 lines of the first table, however, all tables can be printed using print(tab_pred).

*tab_pred <- ga_tab_predictions(gobjs = g_obj_new,*

* data = mcmc_result$samples)*

*head(tab_pred[[1]])*

 ytd  q0.025  q0.5  q0.975

1  1     2    5   11

2  2     2    5   10

3  3     9  19   30

4  4     19   31   44

5  5     0    3     8

6  6     0    2     5

**Fig 4:**
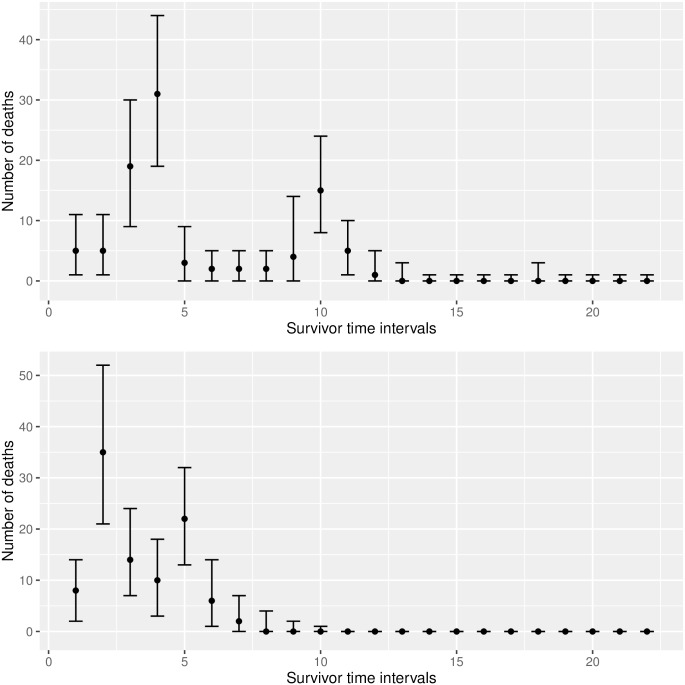
Predictions for 2 fictional (“fake”) experimental setups.

Finally, we create a prediction plot (see Fig 4). The plot shows the medians as well as the quantiles of the predicted survivor counts.

*plot_pred <- ga_plot_predictions(gobjs = g_obj_new,*

* data = mcmc_result$samples)*

**Fig 5:**
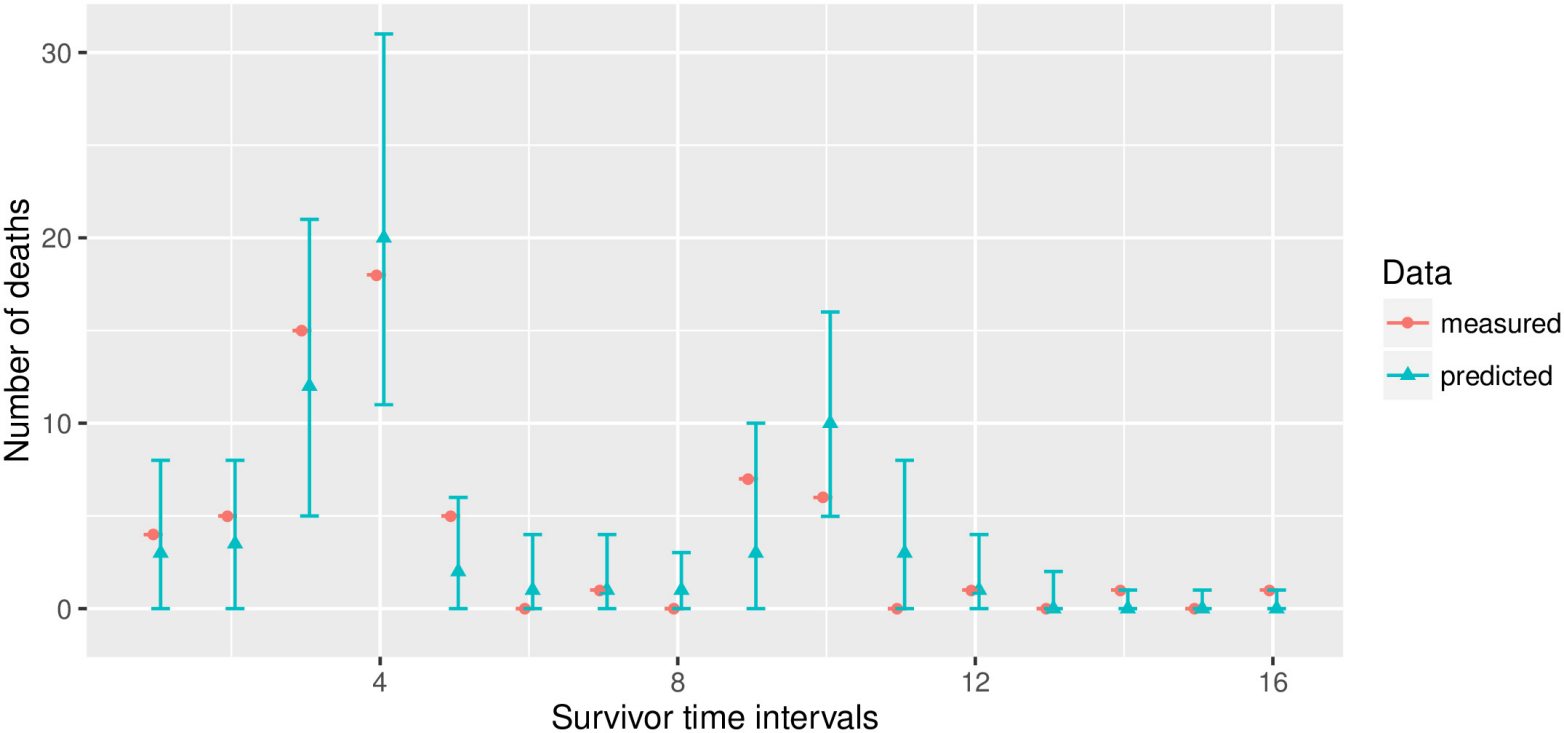
Comparison of a model forecast with fictional (“fake”) data.

The prediction plots show the 95% probability bands and the medians, for the number of deaths that are predicted to occur in each observation window. If measured survivor data is present, it is also possible to add this information to the tables and plots, for validation. We modify our first fictional data set from above and add some (also fictional) survivor data. The tabulation now contains an additional column (“measured”), and the measured data (fictional data, in our example) is added to the plot as well (see Fig 5). Note that each single table can also be saved to text files using the command write.csv().

*g_obj_val <- guts_setup(C = c(99.97824, 0, 103.88, 0, 0, 103.56, 0, 0,*

*   100.58, 96.51, 0, 2.35724),*

*  Ct = c(0, 1.03, 3.01, 4.02, 8, 8.01, 15, 16,*

*   16.01, 17, 18.01, 22.01),*

*  y = c(66, 62, 57, 42, 24, 19, 19, 18, 18, 11, 5, 5, 4, 4, 3, 3, 2),*

*  yt = 0:16)*

*tab_val <- ga_tab_predictions(gobjs = list(g_obj_val),*

*  data = mcmc_result$samples, measured = TRUE)*

*print(tab_val[[1]])*

  ytd   measured    q0.025  q0.5  q0.975

1   1     4  1.000    4     8

2   2     5  0.975    3     8

3   3      15  5.000  12   21

4   4      18   12.000  20   30

5   5     5  0.000    2     6

6   6     0  0.000    1     4

7   7     1  0.000    1     4

8   8     0  0.000    1     4

9   9     7  0.000    3   10

10  10     6  4.000  10   17

11  11     0  0.000    3     8

12  12     1  0.000    1     4

13  13     0  0.000    0     2

14  14     1  0.000    0     1

15  15     0  0.000    0     1

16  16     1  0.000    0     1

*plot_val <- ga_plot_predictions(gobjs = list(g_obj_val),*

* data = mcmc_result$samples, measured = TRUE)*

### Example Code and Further Development

The complete code used throughout the presentation here is available with the paper (see S1 Script and S1 Data). We believe that the conciseness of our code and the application of our self-created wrapper functions make the procedures very easy to understand and to reproduce. However, with more expertise in R, users can easily alter our code and produce their own output. For instance, the plotting routines provided by the R package ggplot2 [38] are very powerful and allow for rich-featured ready-to-publish graphics. We also encourage users to try out different optimisation and inference routines.

Future development will focus on the implementation of more distributions as well as further performance improvements. Users of our R package GUTS are encouraged to provide ideas, feedback or feature requests to the authors and the R GUTS user community, or to contribute actively to further development as a co-developer. The best way to communicate is via the mailing list of the package (guts-users@lists.r-forge.r-project.org). The development home page of our R package GUTS can be found on R-Forge (https://r-forge.r-project.org/projects/guts/).

## Discussion and Future Directions

We discuss the modelling of survival under chemical stress using GUTS [4]. GUTS places the assumptions underlying survival modelling in a consistent mathematical framework, but the calibration has been a challenge. In particular the calibration of toxicodynamic parameters, and the estimation of parametric and predictive uncertainty was still a problem as it required much computational power and time.

GUTS is a survival analysis tool specifically designed to account for time-varying stressors. It is also possible to integrate multiple, independently acting stressors by adding hazard rates [25, 26]. However, most intriguing are the possibilities to better understand underlying mechanisms my meaningful interpretation of the GUTS parameters. We expect that our software facilitates re-analyses of existing survival data as well as asking new research questions in a wide range of sciences. In particular the ability to quickly quantify stressor thresholds in conjunction with dynamic compensating processes, and their uncertainty, is an improvement that complements current survival analysis methods.

## Supporting Information

S1 Script GUTS example R script. Auxiliary R Script for the Paper “Computationally Efficient Implementation of a Novel Algorithm for the General Unified Threshold Model of Survival (GUTS)”.
(R) Click here for additional data file.

S1 Data GUTS example data. Example data for the Paper “Computationally Efficient Implementation of a Novel Algorithm for the General Unified Threshold Model of Survival (GUTS)”.
(TXT)
